# Association between metabolic syndrome and 90-day mortality in acute-on-chronic liver failure patients based on propensity score matching and prognostic model construction

**DOI:** 10.1186/s12876-026-04943-x

**Published:** 2026-05-19

**Authors:** Yitao Hu, Fan Zhang, Fengjiao Zhang, Peizheng Xiang

**Affiliations:** 1 Department of Gastroenterology, The Third People’s Hospital of Yunnan Province, Kunming, Yunnan Province 650011 China; 2 Department of Hepatology and Gastroenterology, The Third People’s Hospital of Kunming, Kunming, Yunnan Province 650041 China

**Keywords:** Metabolic syndrome, Acute-on-chronic liver failure, Mortality, Prediction model

## Abstract

**Background:**

This study aimed to evaluate the association between metabolic syndrome (MetS) and clinical outcomes in patients hospitalized for acute-on-chronic liver failure (ACLF), and to establish a prognostic prediction model incorporating MetS.

**Methods:**

A retrospective cohort study was conducted involving 303 ACLF patients admitted to a tertiary hospital between May 2023 and May 2025. Patients were categorized into two groups based on their MetS status (MetS group vs. non-MetS group). The primary outcome was 90-day all-cause mortality. Propensity score matching (PSM) was employed to balance baseline characteristics. The association was assessed using multivariable Cox regression and logistic regression analyses after adjusting for confounders. Based on the results of the multivariable analysis, a nomogram model for predicting 90-day mortality was constructed. The model’s discriminative ability, calibration, and clinical utility were evaluated in both the training and testing sets using receiver operating characteristic (ROC) curves, calibration curves, and decision curve analysis (DCA).

**Results:**

After PSM, 206 patients (103 in the MetS group and 103 in the non-MetS group) were included in the final analysis. The 90-day mortality rate was significantly higher in the MetS group than in the non-MetS group (49.51% vs. 27.18%, *p* < 0.001). Multivariate Cox regression analysis showed that MetS, age, alanine aminotransferase (ALT), artificial liver support system (ALSS), transfusion times (TT), international normalized ratio (INR), C-reactive protein (CRP) and hepatocellular carcinoma (HCC) were independent predictors of 90-day death risk in patients with ACLF. The nomogram prediction model constructed based on these variables demonstrated excellent discriminative ability in both the training set (area under the curve, AUC = 0.877) and the testing set (AUC = 0.820). The calibration curve showed a high consistency between the predicted probabilities and the actual observations. Decision curve analysis confirmed the model’s favorable net clinical benefit.

**Conclusion:**

MetS is an independent predictor of poor short-term prognosis in patients with ACLF, significantly increasing the risk of mortality. This study established a nomogram prediction model that integrates MetS, which can accurately assess patients’ short-term mortality risk and may assist clinicians in early risk stratification.

**Supplementary Information:**

The online version contains supplementary material available at 10.1186/s12876-026-04943-x.

## Introduction

Metabolic syndrome, a cluster of metabolic disorders including obesity, diabetes, hypertension, and dyslipidemia, has become an increasingly severe global public health issue. Its pathogenesis is complex, involving insulin resistance, chronic inflammation, and lipid metabolism disorders, significantly increasing the risk of cardiovascular disease, type 2 diabetes, and multi-organ dysfunction [1, 2]. The core pathological change of MetS is hepatic steatosis, namely nonalcoholic fatty liver disease (NAFLD), which can progress to nonalcoholic steatohepatitis (NASH), liver fibrosis, and even cirrhosis, serving as a crucial trigger for liver failure [3, 4]. NAFLD is closely related to MetS, jointly driving the progression of liver injury, especially in obese and diabetic patients, where its incidence and severity are significantly increased [5, 6].

ACLF characterized by rapid deterioration of liver function and multi-organ failure, leading to extremely high short-term mortality [7, 8]. In the pathogenesis of ACLF, systemic inflammatory response and multi-organ dysfunction are considered key factors contributing to disease progression and poor prognosis [9, 10]. Furthermore, ACLF patients often exhibit hormonal abnormalities, such as significantly decreased serum testosterone and increased estrogen in male patients, changes closely related to the degree of liver damage and prognosis [11].

Growing evidence suggests that MetS can promote the occurrence and progression of ACLF through various mechanisms, adversely affecting the prognosis of ACLF patients [12–14]. Potential mechanisms include chronic low-grade inflammation, oxidative stress, exacerbation of hepatic microcirculatory disturbances, and multi-organ dysfunction [1, 12, 13, 15–19]. However, despite these potential links, the relationship between MetS and the short-term prognosis of ACLF patients remains understudied and poorly understood.

In summary, understanding the impact of MetS on the early prognosis of ACLF is of significant importance. This will help guide clinical risk assessment and the development of individualized treatment strategies, ultimately improving patient survival rates and quality of life.

## Patients and methods

### Study participants

This retrospective cohort study was conducted at Kunming Third People’s Hospital, a tertiary care hospital. We included patients discharged between May 1, 2023, and May 31, 2025, with a primary diagnosis of ACLF. Patients were excluded if they met any of the following criteria: (1) insufficient basis for ACLF diagnosis; (2) underwent liver transplantation; (3) missing key clinical data; (4) presence of other serious comorbid conditions. Notably, patients with moderate to massive ascites and HCC at Barcelona Clinic Liver Cancer (BCLC) stage C or D were initially excluded from enrollment. This was to eliminate the confounding influence of ascites volume on body mass index (BMI) calculation and the subsequent diagnosis of MetS. This study was conducted in accordance with the Declaration of Helsinki and was approved by the Ethics Committee of The Third People’s Hospital of Kunming (Approval Number: KSYXLL202601-009). Given the retrospective nature of this study and the use of anonymized patient data, the requirement for informed consent was waived by the Institutional Review Board (IRB) of The Third People’s Hospital of Kunming.

### Data collection

Demographic data, comorbidities, laboratory results, artificial liver treatment status, and transfusion history were extracted from electronic medical records. The primary outcome was 90-day all-cause mortality. Survival data were obtained from medical records, supplemented by telephone follow-up when necessary.

### Outcome variable: ACLF

The diagnosis of ACLF was based on standardized clinical and laboratory criteria, requiring the simultaneous fulfillment of: (1) with a background of chronic liver disease (chronic hepatitis or cirrhosis); (2) serum total bilirubin exceeding 10 times the upper limit of normal, or a daily increase > 17.1 µmol/L; (3) prothrombin activity (PTA) ≤ 40%, or INR ≥ 1.5. All diagnoses were retrospectively confirmed by two attending physicians who independently reviewed medical records and laboratory data. Discrepancies were resolved through discussion, following a standardized protocol to ensure consistency.

ACLF in this study was diagnosed following the 2018 Chinese liver failure guideline [20]. The 2024 updated guideline abandoned fixed bilirubin cut-offs for subjective progressive elevation, which may cause diagnostic variability; thus, the 2018 clear quantitative criteria were retained to ensure objective and consistent enrolment.

The 2018 Chinese guideline set bilirubin at > 10×ULN or a daily rise > 17.1 µmol/L, versus ≥ 5 mg/dL (APASL) [21] and 12 mg/dL (EASL-CLIF) [22]. All criteria adopted INR ≥ 1.5 or PTA ≤ 40% as the core coagulation threshold. The 2018 Chinese and APASL definitions rely solely on severe liver dysfunction without requiring extrahepatic organ failure, while EASL-CLIF emphasizes concomitant multiple extrahepatic organ failures for diagnosis and stratification.

### Exposure variable: MetS

Based on internationally recognized definitions of MetS, previous studies [23–25], and Asian population-specific criteria, MetS was diagnosed in this study if at least three of the following five items were satisfied: (1) Obesity: BMI ≥ 25 kg/m²; (2) Hyperglycemia: fasting blood glucose ≥ 6.1 mmol/L, 2‑h postprandial blood glucose ≥ 7.8 mmol/L, or established diabetes mellitus; (3) Hypertension: systolic/diastolic blood pressure ≥ 130/85 mmHg or receipt of antihypertensive treatment; (4) Hypertriglyceridemia: serum triglycerides (TG) ≥ 1.7 mmol/L; (5) Reduced HDL-C: high-density lipoprotein cholesterol (HDL-C) < 1.04 mmol/L.

The widely accepted global MetS criteria adopt identical cutoff values for blood pressure, TG and HDL-C as used in the present study, but apply waist circumference to define abdominal obesity. The International Diabetes Federation (IDF) definition assumes central obesity at a BMI > 30 kg/m², without the necessity of waist circumference measurement [26]. Given ethnic differences in body composition, the present study adopted the Asian-specific BMI cutoff of ≥ 25 kg/m² to define obesity [23]. This approach ensured population suitability while maintaining consistency with international diagnostic thresholds for metabolic and hypertensive components.

### Covariates

The following baseline variables were collected as covariates: age (years); gender (male/female); hemoglobin (HB); total bilirubin (TBIL); aspartate aminotransferase (AST); ALT; creatinine (Cr); serum sodium (Na); INR; white blood cell count (WB); platelet count (PLT); CRP; TT; MELD-Na score; presence of HCC; presence of hepatorenal syndrome; presence of hepatic encephalopathy; whether to perform artificial liver support therapy; and etiology of chronic liver diseases. This evidence suggests an association with the short-term prognosis of patients with ACLF. Data collection was based on the initial laboratory results at admission.

### Statistical analysis

The overall completeness of the dataset was high, with missing data for all variables being < 5%. Missing data for covariates were handled using multiple imputation by chained equations (MICE). To reduce potential bias arising from baseline differences between the MetS and non-MetS groups, PSM was applied. Propensity scores were estimated via logistic regression, incorporating covariates such as demographic data, comorbidities, laboratory results, treatment methods, and MELD-Na scores. A 1:1 nearest-neighbor matching without replacement was performed between MetS and non-MetS patients, with a caliper width set at 0.2 times the standard deviation of the logit of the propensity score. To assess the balance of covariates after matching, standardized mean differences (SMD) were calculated for all baseline characteristics. The final analysis included 206 patients (103 matched pairs).

Continuous variables conforming to a normal distribution are presented as mean ± standard deviation and compared using the t-test. Variables with a non-normal distribution are expressed as median and interquartile range (P25, P75) and compared using the Mann–Whitney U test. Categorical variables were compared using the chi-square test or Fisher’s exact test. Time-to-event data (90-day mortality) were analyzed using Kaplan–Meier curves and multivariable Cox proportional hazards regression. To enhance the robustness of the results, subgroup analyses were conducted to evaluate the association between MetS and 90-day mortality in patients stratified by treatment intensity, age, sex, and MELD-Na score. After multivariable Cox regression analysis, we identified eight independent risk factors for 90-day mortality in patients with ACLF. Given that our model aimed to early identify ACLF patients with poor prognosis, ALSS and TT were regarded as therapeutic variables rather than baseline predictors. Accordingly, these two variables were excluded from the final predictive model, leaving six independent predictors incorporated into the nomogram. Model discrimination was assessed using ROC and the AUC. Calibration was tested using the bootstrap method (500 repetitions). Clinical utility was evaluated through DCA. Hazard ratios (HR) and their 95% confidence intervals (CI) were calculated. All statistical analyses were performed using R software (version 4.5.1), with packages including survival, MatchIt, regplot, rms, shapr, rmda, and shapr. A p value < 0.05 was considered statistically significant.

## Results

### Baseline characteristics after propensity score matching

**Fig. 1:**
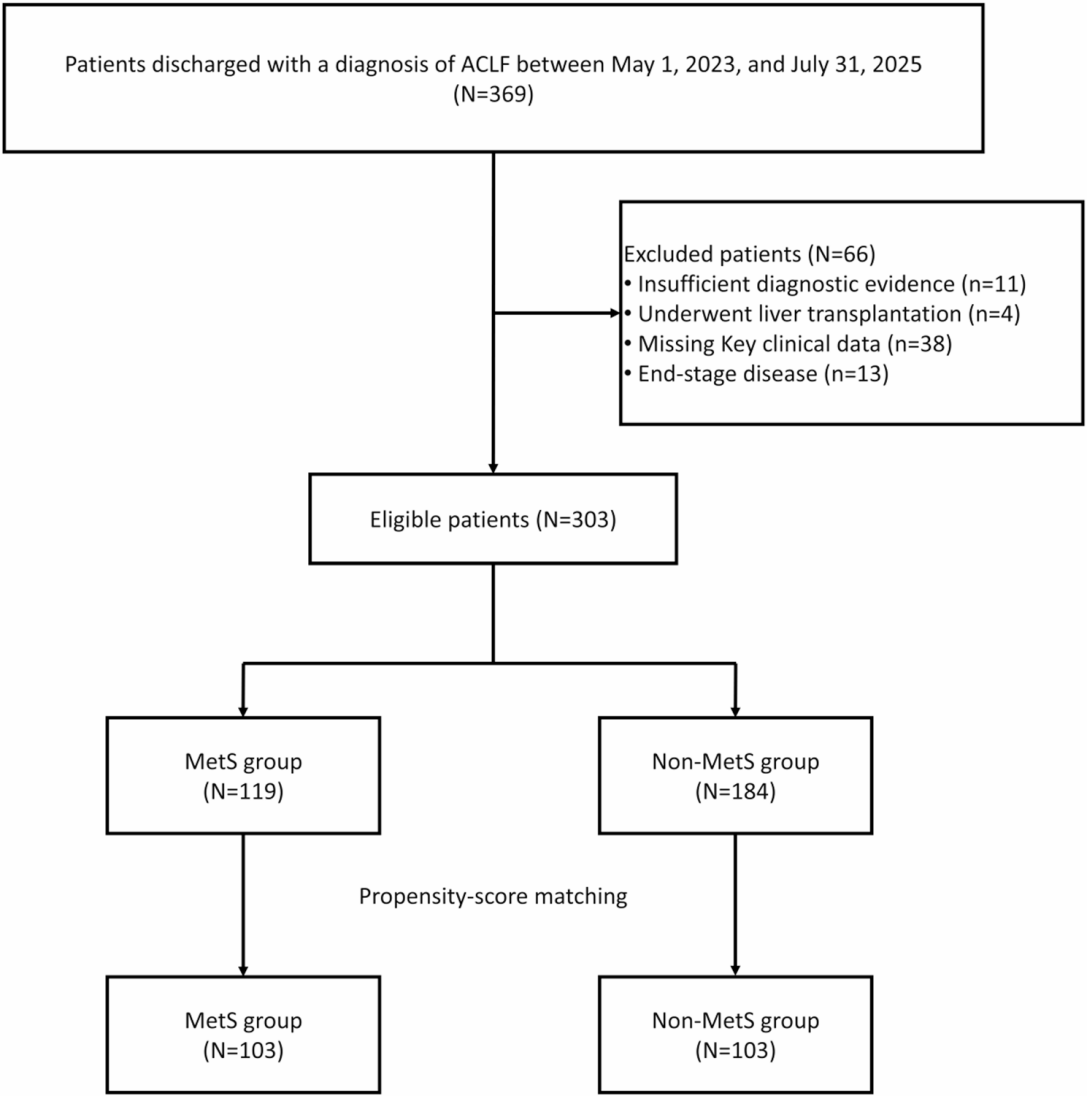
Research flowchart of the study

A total of 369 patients diagnosed with ACLF were initially enrolled. After excluding 66 cases due to insufficient diagnostic basis (*n* = 11), liver transplantation (*n* = 4), missing key clinical data (*n* = 38), and end-stage disease (*n* = 13), the final analysis included 303 participants. Among them, 119 (39.3%) were identified as having MetS. PSM yielded 103 matched pairs, resulting in 206 patients for subsequent comparisons (Fig. 1). As shown in Table 1, all SMDs after matching were less than 0.1, indicating that matching significantly improved the balance of baseline characteristics between groups.

**Table 1:** Baseline characteristics of the study population

	MetS group	Non-MetS group	*P*value	SMD	Balanced?
N	103	103			
Age, years	51.21 ± 11.37	52.41 ± 13.36	0.490	0.089	Yes
HB	128.25 ± 29.05	128.31 ± 29.79	0.989	0.002	Yes
Meld-Na	21.76 ± 6.00	21.59 ± 6.79	0.848	-0.025	Yes
TT	1.00 (0.00, 4.50)	0.00 (0.00, 3.50)	0.540	-0.061	Yes
TBIL	234.60 (189.85, 323.25)	214.80 (182.20, 320.55)	0.546	-0.046	Yes
AST	233.00 (100.50, 551.00)	169.00 (112.00, 359.00)	0.228	-0.032	Yes
ALT	122.00 (50.00, 609.50)	131.00 (50.50, 470.00)	0.969	-0.005	Yes
Cr	68.00 (53.50, 81.00)	63.00 (54.00, 87.50)	0.859	0.049	Yes
Na	135.40 (132.35, 137.95)	135.10 (132.35, 138.10)	0.947	0.057	Yes
CRP	13.64 (8.43, 39.17)	14.52 (10.35, 33.07)	0.729	0.012	Yes
INR	1.76 (1.57, 2.03)	1.65 (1.54, 1.98)	0.201	-0.028	Yes
WB	7.09 (4.94, 9.96)	7.01 (5.66, 9.71)	0.553	-0.044	Yes
PLT	97.00 (77.50, 160.00)	113.00 (77.00, 156.00)	0.848	0.025	Yes
Gender, n (%)			0.853		
Female	18 (17.48)	17 (16.50)		-0.026	Yes
Male	85 (82.52)	86 (83.50)		0.026	Yes
HCC, n (%)			1.000		
No	83 (80.58)	83 (80.58)		0.000	Yes
Yes	20 (19.42)	20 (19.42)		0.000	Yes
HRS, n (%)			0.818		
No	93 (90.29)	92 (89.32)		-0.031	Yes
Yes	10 (9.71)	11 (10.68)		0.031	Yes
HE, n (%)			0.761		
No	73 (70.87)	71 (68.93)		-0.042	Yes
Yes	30 (29.13)	32 (31.07)		0.042	Yes
ALSS, n (%)			0.780		
No	52 (50.49)	54 (52.43)		0.039	Yes
Yes	51 (49.51)	49 (47.57)		-0.039	Yes
Reason, n (%)			0.932		
Viral	69 (66.99)	65 (63.11)		-0.080	Yes
Alcoholic	14 (13.59)	14 (13.59)		0.000	Yes
Drug-induced	9 (8.74)	9 (8.74)		0.000	Yes
Autoimmune	1 (0.97)	2 (1.94)		0.070	Yes
Multifactorial	10 (9.71)	13 (12.62)		0.088	Yes

*Abbreviations SMD* standardized mean differences, *MetS* metabolic syndrome, *HB * hemoglobin, *Meld-Na *model for end-stage liver disease-sodium, *TT* transfusion times, *TBIL* total bilirubin, *AST * aspartate aminotransferase, *ALT * alanine aminotransferase, *Cr * creatinine, *Na* sodium, *CRP * C-reactive protein, *INR * international normalized ratio, *WB * white blood cell, *PLT* platelets, *HCC * hepatocellular carcinoma, *HRS* hepatorenal syndrome, *HE* hepatic encephalopathy, *ALSS* artificial liver support system

### Prognostic impact of MetS on 90-day all-cause mortality

**Fig. 2:**
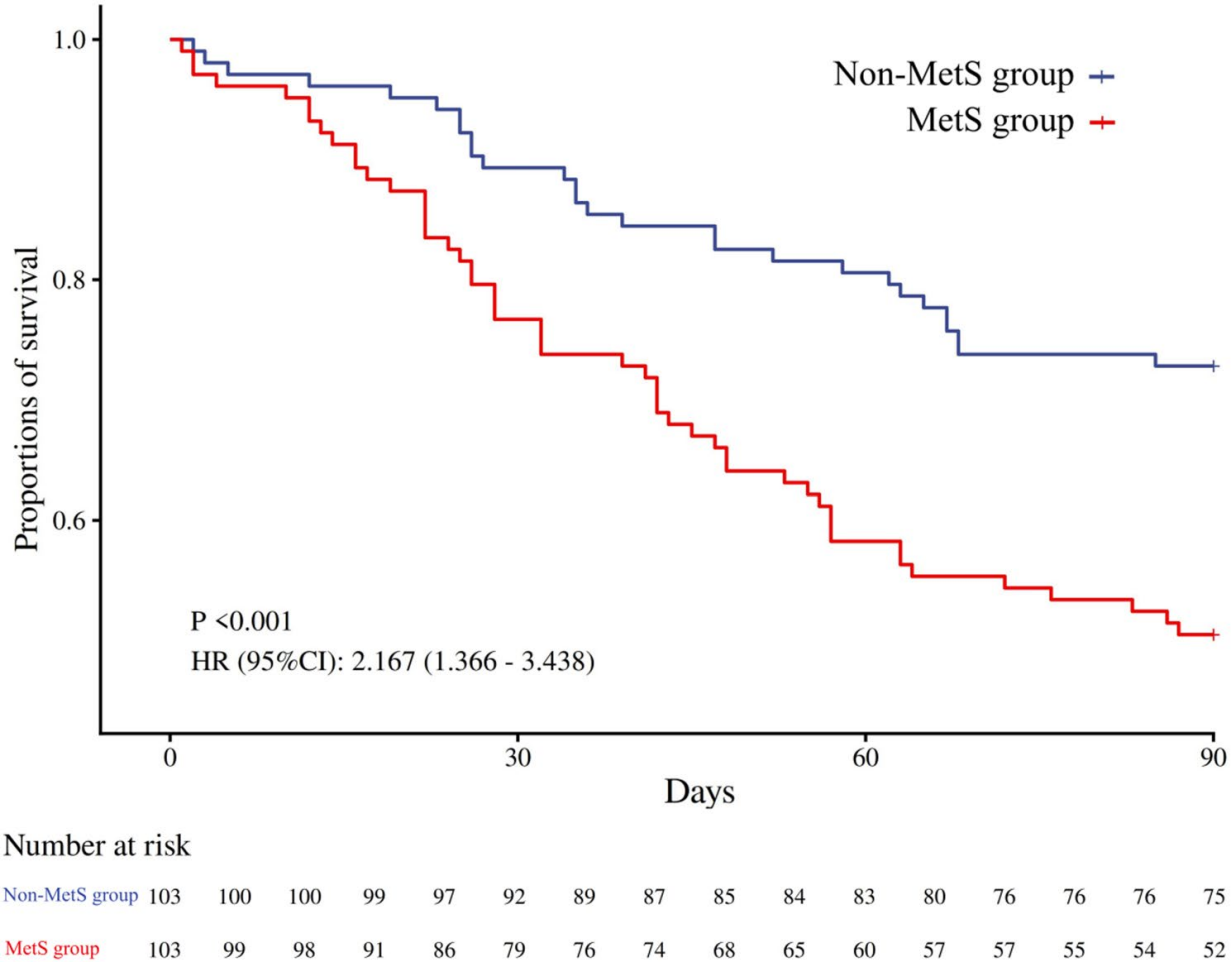
Kaplan-Meier survival curves for 90-day all-cause mortality in MetS and non-MetS groups

The 90-day mortality was 49.51% in the MetS group and 27.18% in the non-MetS group (*p* < 0.001). Kaplan-Meier survival analysis (Fig. 2) showed that patients with MetS had a significantly higher risk of 90-day all-cause mortality compared to those without MetS (HR = 2.167, 95% CI: 1.366–3.438; *p* < 0.001). Multivariable Cox proportional hazards analysis, after adjusting for potential confounders, indicated that MetS was independently associated with a 3.12-fold increased mortality risk compared to the non-MetS group (95% CI: 1.91–5.10; *p* < 0.001) (Table 2).

**Table 2:** Univariate and Multivariate COX regression for exploring the association of MetS status with 90-day mortality

	Univariate analysis			Multivariate analysis		
	HR	95%CI	*P* value	HR	95%CI	*P* value
Age	1.04	1.02 ~ 1.06	< 0.001	1.04	1.01 ~ 1.06	0.003
Gender						
Female	1.00	Reference				
Male	0.82	0.47 ~ 1.44	0.497			
TBIL	1.01	1.01 ~ 1.01	0.043	1.00	1.00 ~ 1.00	0.877
AST	0.99	0.99 ~ 0.99	0.028	1.00	1.00 ~ 1.00	0.594
ALT	0.99	0.99 ~ 0.99	< 0.001	0.99	0.99 ~ 0.99	0.025
Cr	1.01	1.01 ~ 1.01	< 0.001	1.00	1.00 ~ 1.01	0.507
Na	0.91	0.87 ~ 0.95	< 0.001	0.97	0.92 ~ 1.02	0.183
CRP	1.01	1.01 ~ 1.01	< 0.001	1.01	1.01 ~ 1.02	0.029
INR	2.38	1.59 ~ 3.57	< 0.001	5.64	2.91 ~ 10.95	< 0.001
WB	1.04	1.01 ~ 1.08	0.012	1.03	0.99 ~ 1.07	0.204
HB	0.99	0.98 ~ 0.99	< 0.001	1.00	0.99 ~ 1.01	0.729
PLT	1.00	0.99 ~ 1.00	0.293			
Meld-Na	1.11	1.08 ~ 1.15	< 0.001	1.04	0.97 ~ 1.11	0.269
Mets						
No	1.00	Reference		1.00	Reference	
Yes	2.17	1.37 ~ 3.44	0.001	3.12	1.91 ~ 5.10	< 0.001
Reason						
Viral	1.00	Reference				
Alcoholic	0.97	0.52 ~ 1.80	0.918			
Drug-induced	0.57	0.23 ~ 1.42	0.226			
Autoimmune	0.00	0.00 ~ Inf	0.995			
Multifactorial	0.45	0.18 ~ 1.13	0.089			
HCC						
No	1.00	Reference		1.00	Reference	
Yes	3.22	2.02 ~ 5.14	< 0.001	2.25	1.20 ~ 4.25	0.012
HRS						
No	1.00	Reference		1.00	Reference	
Yes	2.67	1.47 ~ 4.85	0.001	2.04	0.91 ~ 4.56	0.082
HE						
No	1.00	Reference				
Yes	1.32	0.83 ~ 2.10	0.246			
ALSS						
No	1.00	Reference		1.00	Reference	
Yes	0.47	0.29 ~ 0.74	0.001	0.55	0.32 ~ 0.95	0.031
TT	0.89	0.82 ~ 0.96	0.005	0.76	0.68 ~ 0.85	< 0.001

*Abbreviations HR* hazard ratios, *CI* confidence intervals, *TBIL* total bilirubin, *AST * aspartate aminotransferase, *ALT* alanine aminotransferase, *Cr* creatinine, *Na* sodium, *CRP * C-reactive protein, *INR* international normalized ratio, *WB* white blood cell, *HB* hemoglobin, *PLT* platelets, *Meld-Na* model for end-stage liver disease-sodium, *MetS* metabolic syndrome, *HCC* hepatocellular carcinoma, *HRS* hepatorenal syndrome, *HE* hepatic encephalopathy, *ALSS* artificial liver support system, *TT* transfusion times

### Subgroup analysis of the association between MetS and 90-day prognosis

**Fig. 3:**
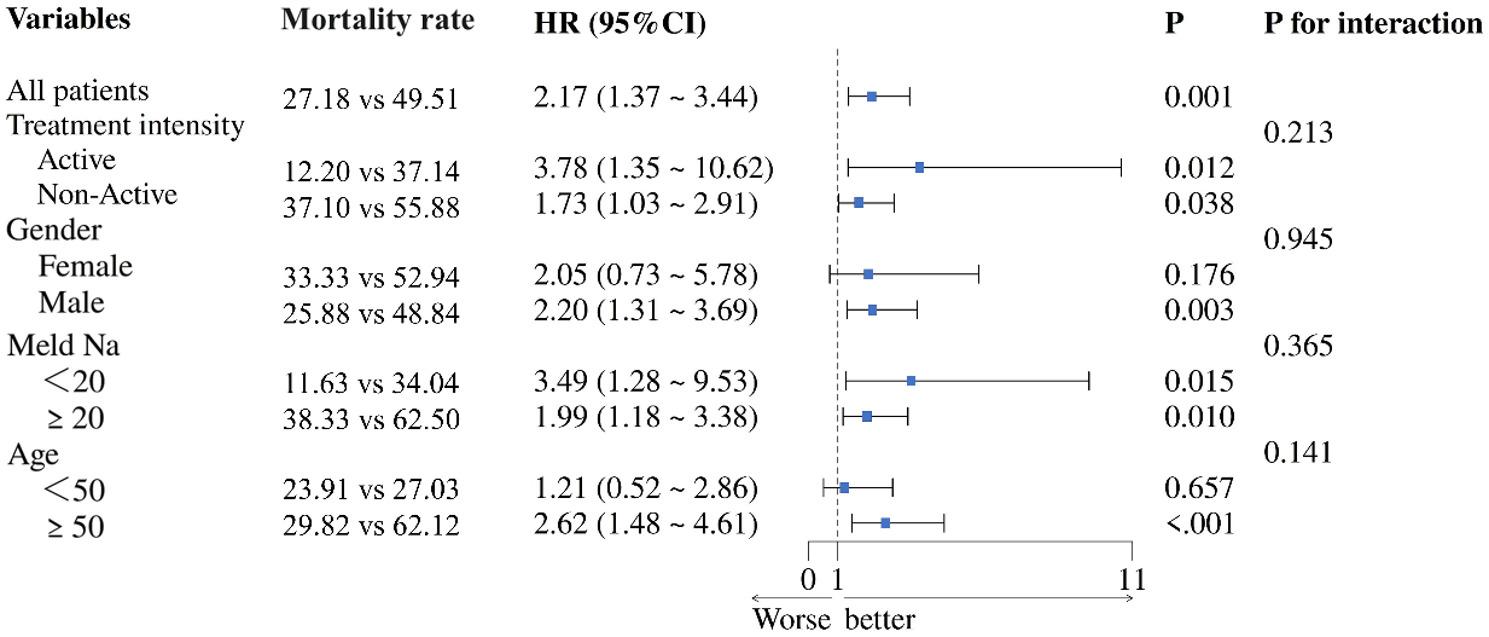
Forest plot of subgroup analyses for the association between MetS and 90-day mortality

To test the robustness and potential heterogeneity of the association between MetS and 90-day prognosis in ACLF patients, we performed stratified analyses by treatment intensity, age, sex, and disease severity (MELD-Na score). Patients with ≤ 1 session of ALSS were categorized into the non-active group, while those with more than one session were defined as the active group. Subgroup analysis results showed that the trend of MetS increasing the risk of poor prognosis remained consistent across most subgroups (HR > 1 for all subgroups). Specifically, MetS served as an independent prognostic risk factor in males, patients aged ≥ 50 years, and across subgroups stratified by MELD-Na score and treatment intensity (all *P* < 0.05). Detailed HR and their 95% CI for each subgroup are shown in Fig. 3 (forest plot). The figure shows that, except for the female and age < 50 subgroups, the confidence intervals for all other subgroups did not include 1, and the point estimates (HR) were all greater than 1, All interaction P values were greater than 0.05, indicating that the effect of MetS on 90-day mortality did not differ significantly across these subgroups.

### Develop and evaluate nomograms for ACLF patients prognosis outcomes

**Fig. 4:**
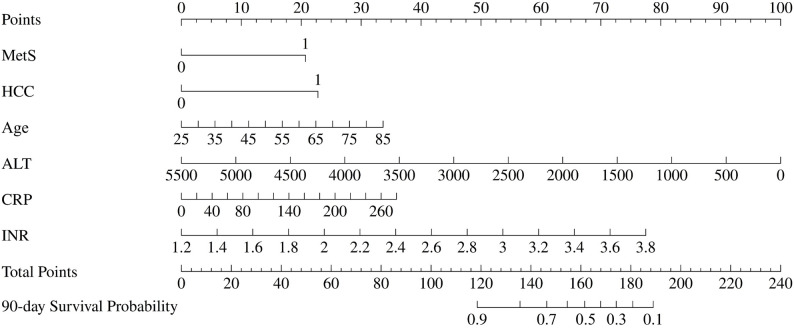
Nomogram for predicting the 90-day mortality risk in ACLF patients

**Fig. 5:**
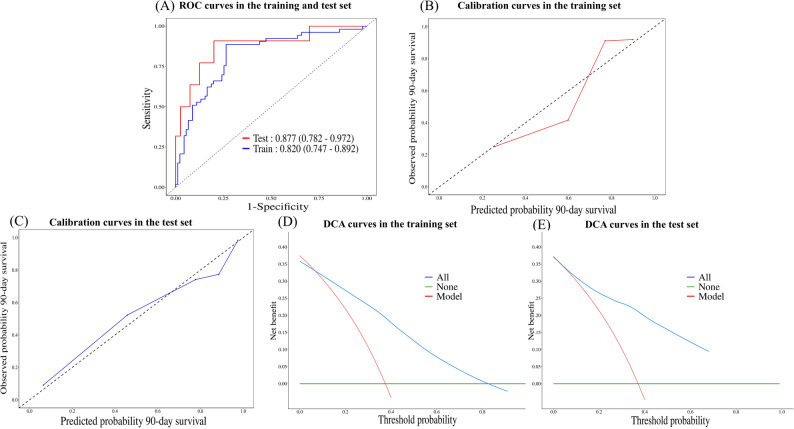
Internal validation of the predicting model: (**A**) ROC curves, (**B**, **C**) Calibration curves, (**D**, **E**) Decision curve analysis

**Fig. 6:**
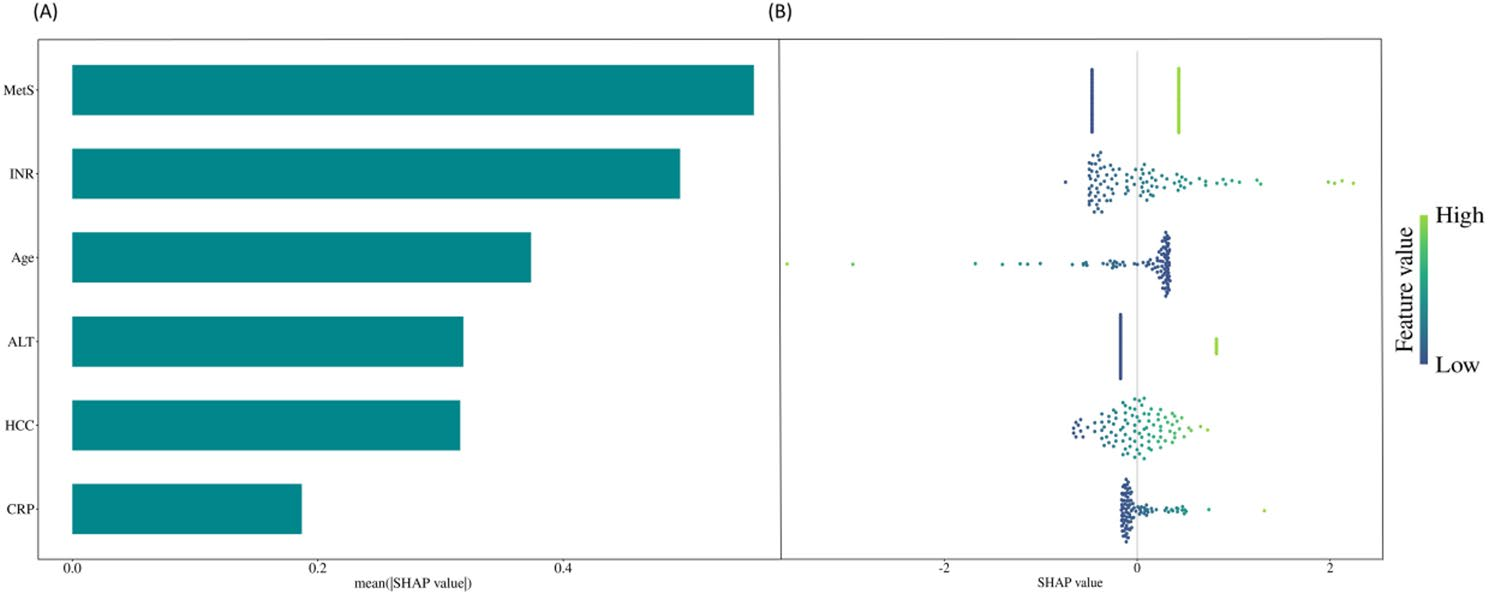
SHAP analysis for the prediction model: (**A**) A bar summary of the most important features according to the SHAP values, (**B**) Summary and explanation of the most influential features

Based on the results of the multivariable Cox analysis, six indicators were incorporated: MetS, Age, ALT, CRP, INR, presence of HCC. Using the rms package in R software, we created a visual nomogram to represent the prediction model (Fig. 4). Each variable in the nomogram is assigned a score, which are summed to obtain a total score used to assess the 90-day mortality risk for ACLF patients. The data were randomly split into a 70% training set and a 30% validation set for internal validation of the model. In the training set, a total of 55 deaths were recorded. The event per variable (EPV) value of this six-variable predictive model was approximately 9, which exceeded the acceptable minimum threshold of EPV ≥ 5 and was close to the optimal criterion of EPV ≥ 10. This favorable EPV level indicated an adequate sample size and ensured the statistical stability and credibility of the constructed nomogram, minimizing the risk of overfitting. ROC curve analysis (Fig. 5A) demonstrated that the AUC in the training and validation cohorts were 0.877 and 0.820, respectively, indicating the model’s ability to accurately distinguish patient mortality risk with good generalizability. Calibration curves were generated using the bootstrap method (500 replications), Calibration curves (Fig. 5B and C) demonstrated a high degree of agreement between the model-predicted mortality probabilities and the actual observed probabilities, confirming the accuracy and reliability of its predictions. DCA (Fig. 5D and E) confirmed that the nomogram provided a good net clinical benefit across a wide range of threshold probabilities, supporting its practical value as a clinical decision aid. Based on SHAP analysis (Fig. 6A), MetS and INR were identified as the features with the highest contribution to the model’s predictions. High values for INR and MetS showed strong positive contributions to individual sample predictions (Fig. 6B), highlighting the central role of metabolic disorders in ACLF prognosis and providing important targets for clinical intervention.

## Discussion

This study aimed to investigate the impact of metabolic syndrome on the 90-day mortality risk in patients with ACLF and to construct a corresponding prediction model. By analyzing the epidemiological characteristics and clinical outcomes of MetS in ACLF patients, we hope to provide a more precise risk assessment tool for clinical practice. The results indicate that MetS significantly affects the survival prognosis of ACLF patients. Compared to patients without MetS, those with MetS had a 3.12-fold higher risk of 90-day all-cause mortality. This association persisted even after adjusting for confounders such as age, sex, comorbidities, treatment methods, and laboratory indicators in multivariable analysis, establishing MetS as an independent prognostic factor for ACLF patients. Given the inherently high mortality of ACLF, accurately identifying high-risk patients can provide crucial guidance for clinical management.

Our study found that transfusion times acted as a protective factor for the 90-day prognosis of patients with ACLF, which contradicts the common clinical consensus that critically ill patients require more blood transfusions. This phenomenon was mainly attributed to selection bias. Patients with stable conditions tended to receive aggressive treatment including repeated blood transfusions and ALSS, while critically ill patients had lower treatment willingness. Furthermore, some critically ill patients died early before receiving adequate transfusions, leading to fewer transfusion times in this high-risk population. Such inherent selection bias may overestimate the protective correlation of transfusions. Thus, this paradoxical protective effect should be interpreted with caution and does not represent the genuine therapeutic benefits of blood transfusion. Notably, subgroup analysis demonstrated that MetS consistently increased the risk of mortality regardless of treatment intensity.

The insignificant prognostic effect of MetS observed in female and younger subgroups may be primarily attributed to sample limitations. The female population only accounted for approximately 17% of the overall cohort, resulting in insufficient statistical power to detect potential associations. For patients aged < 50 years, this subgroup generally exhibited stronger liver compensatory reserve, lower systemic inflammatory levels, and fewer underlying chronic comorbidities. These favorable physiological characteristics may mitigate the adverse impact of MetS on short-term mortality, thereby weakening its statistical significance in younger individuals. Accordingly, we caution against interpreting these non-significant results as evidence of a null effect. Further large-scale, well-powered studies stratified by gender and age are warranted to validate the subgroup-specific influence of MetS in ACLF populations.

Metabolic syndrome, as a cluster of metabolic abnormalities, is often accompanied by insulin resistance, dyslipidemia, obesity, and hypertension, with particularly significant impacts on liver function. The oxidative stress triggered by metabolic disorders is a key mechanism of hepatocyte injury and apoptosis. Insulin resistance leads to abnormal fatty acid metabolism, causing excess fatty acids to accumulate in the liver and promoting lipid peroxidation, which generates large amounts of reactive oxygen species (ROS). Excessive ROS accumulation disrupts intracellular redox balance, causing oxidative damage to lipids, proteins, and DNA, thereby activating various apoptotic signaling pathways [27, 28].

In ACLF patients with comorbid MetS, the risk of complications such as renal failure, infection, and circulatory failure increases significantly, leading to a marked rise in short-term mortality [29, 30]. The systemic inflammatory response and immune dysfunction induced by MetS make patients more susceptible to multi-organ dysfunction, particularly damage to the renal and circulatory systems, which become important factors contributing to poor prognosis [31, 32]. Further mechanistic studies reveal that ACLF patients exhibit significant immunometabolic disturbances in hepatic and peripheral blood mononuclear cells, leading to immune system imbalance and severe inflammatory responses. Metabolic abnormalities promote functional reprogramming of immune cells, particularly an increase in the immunosuppressive phenotype of liver macrophages, contributing to an immunodeficient state that increases infection risk and organ failure [33, 34]. Furthermore, the abnormal accumulation of fatty acids and their metabolites related to MetS in the liver promotes the release of inflammatory mediators, exacerbating the hepatic microenvironment and accelerating the progression to liver failure [35].

It is well-known that bleeding is also a significant cause of death in ACLF patients. The core features of MetS—chronic low-grade inflammation and oxidative stress—can directly damage vascular endothelial function, leading to endothelial cell apoptosis and disruption of vascular wall integrity [36]. Endothelial dysfunction not only promotes atherosclerosis but may also increase microvascular fragility, making vessels prone to rupture and bleed under blood pressure fluctuations or local factors. Secondly, platelet dysfunction is common in MetS patients. Insulin resistance and an inflammatory environment may simultaneously cause qualitative platelet defects, such as decreased reactivity or exhaustion after overactivation, leading to insufficient function when effective hemostasis is required [37]. Additionally, MetS is often associated with elevated levels of plasminogen activator inhibitor-1 (PAI-1). While this primarily leads to inhibited fibrinolysis and a prothrombotic tendency, in certain local microenvironments, persistent inflammation and metabolic disturbances may also contribute to hemostatic insufficiency through other pathways, such as coagulation factor consumption or abnormal anticoagulant production [37].

In summary, MetS significantly increases the risk of ACLF by exacerbating the metabolic burden and immune imbalance in the liver. When ACLF patients have comorbid MetS, the risks of infection and bleeding are elevated, leading to a significant increase in short-term mortality.

In our study, multivariable analysis revealed independent relationships between 90-day mortality risk and MetS, CRP, INR, ALT, Age, among others. This is consistent with the conclusions of the aforementioned studies. Elevated ALT levels are directly related to the degree of hepatocyte injury, while age indirectly affects prognosis by influencing liver regenerative capacity and metabolic function. INR reflects the massive loss of functional hepatocytes, and severe infections or sepsis exacerbating coagulation disorders (such as disseminated intravascular coagulation, DIC) can further worsen INR. Furthermore, it is noteworthy that in this study, SHAP analysis showed varying degrees of contribution from different components to the model’s predictions. The significant contribution of MetS as a key feature underscores the central role of metabolic disorders in ACLF prognosis.

The limitations of this study are primarily reflected in the relatively small sample size and the single data source. Although we included 303 ACLF patients, the sample size may still be insufficient to fully represent the characteristics of a broader population, potentially affecting the external generalizability of the results. Additionally, the study was conducted at a single hospital, which may introduce selection bias and limit the applicability of the findings in different healthcare settings. On the other hand, while an effective prediction model was established, the lack of long-term follow-up data prevents us from evaluating the role of MetS in the long-term prognosis of ACLF patients.

Another notable limitation of this study pertains to the confounding effect of ACLF on the assessment of metabolic syndrome components, with particular respect to BMI and triglyceride levels. Acute hepatic decompensation is typically accompanied by fluid retention, visceral congestion, and robust systemic inflammation, which can transiently perturb anthropometric and biochemical metabolic parameters and hinder the accurate evaluation of baseline metabolic status. Although patients with moderate to massive ascites were excluded to mitigate such interference, subtle fluid overload and persistent inflammatory stress in critically ill ACLF patients may still subtly distort metabolic indicators. We acknowledge that these acute illness-induced fluctuations inevitably introduce measurement bias, potentially masking the true baseline prevalence of MetS among HCC patients. Accordingly, well-designed prospective studies incorporating pre-admission metabolic profiling and longitudinal dynamic monitoring of metabolic parameters throughout hospitalization are urgently required to validate our findings and further elucidate the causal interplay between metabolic derangements and clinical outcomes in this vulnerable population.

This study preliminarily illustrated the potential role of MetS in predicting the 90-day mortality risk among patients with ACLF, and established a visualized predictive model with acceptable predictive performance. The model may assist in identifying patients at high mortality risk and provide a preliminary reference for individualized therapeutic strategies. Considering the single-center design, relatively limited sample size, and only internal validation performed in this study, further multicenter investigations with larger patient cohorts are warranted to validate the generalizability of this model. Additionally, targeted interventions aimed at metabolic abnormalities may represent a promising direction for optimizing prognostic outcomes in patients with ACLF.

## Conclusion

This study confirmed that MetS is associated with short‑term prognosis in patients with ACLF. ACLF patients complicated with MetS exhibited an increased 90‑day mortality risk, and interventions targeting metabolic syndrome may improve patient prognosis.

## Supplementary Information

Supplementary Material 1.

## Data Availability

The data collected for this study will be made publicly available. Interested parties can contact the corresponding authors (PZX ) for access.

## Abbreviations

HB: Hemoglobin
Meld-Na: Model for end-stage liver disease-sodium
TT: Transfusion times
TBIL: Total bilirubin
AST: Aspartate aminotransferase
ALT: Alanine aminotransferase
Cr: Creatinine
CRP: C-reactive protein
INR: International normalized ratio
WB: White blood cell
PLT: Platelets
HCC: Hepatocellular carcinoma
HRS: Hepatorenal syndrome
HE: Hepatic encephalopathy
ALSS: Artificial liver support system
