# Supplementary material for: Association between metabolic syndrome and 90-day mortality in acute-on-chronic liver failure patients based on propensity score matching and prognostic model construction
Source: BMC Gastroenterol. 2026 May 19;26:434. doi: 10.1186/s12876-026-04943-x (PMC13352876; doi:10.1186/s12876-026-04943-x)
Supplement: Supplementary file 1 — Supplementary Material 1. [file 12876_2026_4943_MOESM1_ESM.pdf]

昆明市第三人民医院伦理审查委员会伦理审查批件

批件号：KSYXLL202601-009

审查日期	2026 年 01 月 05 日
审查会议地点	NA
研究项目名称	代谢综合征与肝衰竭患者预后的关系的模型构建与验证
项目编号	NA
审查文件	☑ 伦理审查申请表 ☐ 药物审查申请表 ☐ 持续审查申请表<br>☐ 方案修改申请表 ☐ 不良反应报告表 ☐ 不服从行为报告表<br>☐ 研究结束申请表 ☐ 临床研究批准文或资金来源证明、合同<br>☑ 全部研究方案 ☑ 知情同意书 ☐ 知情同意告知信<br>☐ 研究期间发表文章 ☐ 志愿者招募海报或广告 ☐ 电话或口头招募信<br>☐ 问卷或调查表 ☐ 伦理审查委托授权书 ☐ 合作方伦理审查批件<br>☐研究负责人履历 ☐研究组人员培训记录 ☐同类研究进展资料<br>☐ 其他：<br>☐ 临床研究批准文号：
申办者	昆明市第三人民医院
临床研究部门	肝病消化科
研究负责人	向培正
伦理审查方式	☐ 全体会议审查 ☐ 快速审查 ✓ 免除审查
审查委员	
审查决定	☑ 同意临床研究方案<br>☐ 作必要修改后正式同意<br>☐ 作必要修改后重新会议审查<br>☐ 不同意临床研究方案<br>☐ 终止或暂停临床研究方案
伦理审查批件有效期	2026 年 01 月 05 日至 2027 年 01 月 04 日
伦理委员会联系电话	0871-63513914
伦理委员会主席签字	（签名）
伦理委员会盖章	（印章：昆明市第三人民医院伦理委员会）
日期	2026 年 01 月 05 日

提示：研究负责人必须严格使用经审查同意的知情同意书文本和研究方案。如伦理审查批件有效期内不能完成所有的临床研究（包括统计分析），请在本批件失效前一个月，递交持续审查申请。如在审查有效期内完成研究，请递交研究结束报告。研究中发生涉及受试者的任何严重不良事件和/或影响风险收益比的非预期不良事件，应立刻报告伦理审查委员会。任何研究方案、知情同意书的修改包括研究人员的变更，必须递交研究方案修改申请，经伦理审查委员会审查获得批准后执行，任何发生违反本方案的或伦理审查委员会决定的情况，应立刻报告。
